# The Spasmolytic, Bronchodilator, and Vasodilator Activities of *Parmotrema perlatum* Are Explained by Anti-Muscarinic and Calcium Antagonistic Mechanisms

**DOI:** 10.3390/molecules26216348

**Published:** 2021-10-20

**Authors:** Musaddique Hussain, Hazoor Bakhsh, Shahzada Khurram Syed, Malik Saad Ullah, Ali M. Alqahtani, Taha Alqahtani, Afaf A. Aldahish, Talha Bin Emran, Kashif Ur Rehman, Khalid Hussain Janbaz

**Affiliations:** 1 Faculty of Pharmacy, The Islamia University of Bahawulpur, Bahawulpur 63100, Pakistan; kashifurrehman@iub.edu.pk; 2 Faculty of Pharmacy, Bahauddin Zakariya University, Multan 66000, Pakistan; dr.hadi@gmail.com (H.B.); khjanbazz@bzu.edu.pk (K.H.J.); 3 Department of Basic Medical Sciences, School of Health Sciences, University of Management and Technology, Lahore 54000, Pakistan; shahzada.khurram@umt.edu.pk; 4 Department of Pharmaceutical Chemistry, Government College University, Faisalabad 38000, Pakistan; dr.mlksaad@gamil.com; 5 Department of Pharmacology, College of Pharmacy, King Khalid University, Abha 62529, Saudi Arabia; amsfr@kku.edu.sa (A.M.A.); ttaha@kku.edu.sa (T.A.); adahesh@kku.edu.sa (A.A.A.); 6 Department of Pharmacy, BGC Trust University Bangladesh, Chittagong 4381, Bangladesh; talhabmb@bgctub.ac.bd

**Keywords:** acute toxicity, spasmolytic, bronchodilator, vasodilator, Ca^+2^ antagonist, *Parmotremaperlatum*

## Abstract

*Parmotremaperlatum* is traditionally used in different areas of Pakistan to treat gastrointestinal, respiratory, and vascular diseases. This study evaluates the underlying mechanisms for traditional uses of *P. perlatum* in diarrhea, asthma, and hypertension. In vitro pharmacological studies were conducted using isolated jejunum, trachea, and aortic preparations, while the cytotoxic study was conducted in mice. Crude extract of *P. perlatum*(Pp.Cr), comprising appreciable quantities of alkaloids and flavonoids, relaxed spontaneously contracting jejunum preparation, K^+^ (80 mM)-induced, and carbachol (1 µM)-induced jejunum contractions in a concentration-dependent manner similar to dicyclomine and dantrolene. Pp.Cr showed a rightward parallel shift of concentration-response curves (CRCs) of Cch after a non-parallel shift similarto dicyclomine and shifted CRCs of Ca^+2^ to rightward much likeverapamil and dantrolene, demonstrating the coexistence of antimuscarinic and Ca^+2^ antagonistic mechanism. Furthermore, Pp.Cr, dicyclomine, and dantrolene relaxed K^+^ (80 mM)-induced and Cch (1 µM)-induced tracheal contractions and shifted rightward CRCs of Cch similar to dicyclomine, signifying the dual blockade. Additionally, Pp.Cr also relaxed the K^+^ (80 mM)-induced and phenylephrine (1 µM)-induced aortic contraction, similarly to verapamil and dantrolene, suggesting Ca^+2^ channel antagonism. Here, we explored for the first time thespasmolytic and bronchodilator effects of Pp.Crand whether they maybe due to the dual blockade of Ca^+2^ channels and muscarinic receptors, while the vasodilator effect might be owing to Ca^+2^ antagonism. Our results provide the pharmacological evidence that *P. perlatum* could be a new potential therapeutic option to treat gastrointestinal, respiratory, and vascular diseases. Hence, there is a need for further research to explore bioactive constituent of *P. perlatum* as well as further investigation by suitable experimental models are required to further confirm the importance and usefulness of *P. perlatum* in diarrhea, asthma, and hypertension treatment.

## 1. Introduction

Diarrhea is a common gastrointestinal problem characterized by the loss of watery stool often with vomiting and fever, and mild to severe life-threatening dehydration. Dehydration is especially hazardous in children, the elderly, and people with weaker immune systems if left untreated. Diarrhea causes approximately 4–5 million deaths annually throughout the world and 80% of these deaths are reported in developing countries [1,2]. Infectious agents like viruses, bacteria and parasites, various medications, lactose intolerance, artificial sweeteners, surgery, and other digestive disorders cause diarrhea [3]. Treatments options for diarrhea include antibiotics (azithromycin, levofloxacin, and ciprofloxacin), antidiarrheal medication (loperamide, bismuth subsalicylate), probiotics, rehydration, and changing one’s lifestyle and diet.

Asthma is a chronic, non-communicable respiratory illness that affects both children and adults and is characterized by a wide range of respiratory symptoms and airflow limitations [4]. Asthma causes wheezing, shortness of breath, breathlessness, chest tightness, and coughing at night or early in the morning. Asthma is caused by complex gene–environment interactions, resulting in a wide range of clinical manifestations as well as the kind and severity of airway inflammation and remodeling [5]. Globally, 339 million individuals suffer from asthma. Asthma is often under-diagnosed and under-treated, particularly in low- and middle-income countries. People with under-treated asthma can suffer sleep disturbance, tiredness during the day, and poor concentration. Steroids, leukotriene antagonists, short- and long-acting bronchodilators, anticholinergic agents, anti-IL4 and IL-5 treatments, anti-IgE antibody and combination therapies (including inhaled corticosteroid, long-acting bronchodilator, and anti-muscarinic agent) are all common asthma treatments, but the symptoms of asthma are not adequately controlled with currently available therapy.

Hypertension, known as high blood pressure (BP), is a chronic medical condition with elevated BP in the arteries, systolic blood pressure ≥140 mmHg and a diastolic blood pressure ≥90 mmHg (≥140/≥90 mmHg). Persistent hypertension is a primary cause of chronic kidney failure, dementia, and blindness, as well as a risk factor for atherosclerosis, strokes, heart attacks, heart failure, and arterial aneurysm [6,7]. Hypertension accounts for around 16.5 percent of yearly fatalities globally, with the annual death toll expected to reach 23.5 million by 2030 [8]. Many medications are used to regulate BP levels in hypertensive patients, including sympathoplegic agents, diuretics, ACE inhibitors, renin inhibitors, Ca^+2^ antagonists, angiotensin receptor blockers, α- and β-adrenergic blockers, and vasodilators. Unfortunately, 34% hypertension is managed via currently available drugs.

Commercially available drugs fordiarrhea, bronchitis, asthma, airway congestion and hypertension are still with certain shortcomings such as side effects, cost, effectiveness, availability and patient compliance. Alternatively, over the last decade, the use of herbal medicine as a therapeutic method has risen substantially [8] because the plants contain a plethora of phytochemicals that have been shown to protect against a variety of illnesses and diseases while having fewer unwanted side effects and being cost effective [8,9,10]. The development of innovative therapeutic methods for the treatment of diarrhea, bronchitis, asthma, airway congestion, and hypertension has become more essential.

In this regard, various ethno-pharmacological studies have been conducted on isolated smooth muscles preparations [11,12,13,14,15,16,17,18] for the discovery and development of novel, safe, and affordable medicines. In the human body, smooth muscle contraction by the cytosolic Ca^+2^ is important for regulating the normal function of respiratory, gastro intestinal tract (GIT), and vascular system, while malfunctioning of these smooth muscles may lead to airway disorders (asthma), intestinal disorders (spasms and cramps), and cardiovascular disorders (hyper- and/or hypo-tension) [19]. Interestingly, isolated tissue from rabbits, rats or guinea pigs share many anatomical, pharmacological, and physiological similarities of similar human organs [20] and are therefore preferably used because of sensitivity, availability, cost-effectiveness, and reproducibility of results. Plant extracts can coordinate and restore the malfunctioning of smooth muscles.

*Parmotrema perlatum* Huds. is a lichen referred by synonym *Parmelia perlata* (Huds.) Ach. and known by vernacular name of “Stone Flower” and/or “Charila”. *P. perlatum* grows on old trees and walls, widely dispersed in hilly areas of Europe, North and South America, Asia, cultivated in Himalayas and Kashmir [21]. Reproduction of *P. perlatum* occurs by spores and dispersed by wind. Traditionally, *P. perlatum* is being used as decoction as well as powder form (whole plant) with dosage range of 30 to 40 mL of decoction and 1–3 gm of powder. It has a bitter or saline taste, and is used as a spice [21,22].

Previous phytochemical analysis of *P. perlatum* exposed the presence of atranorin, lecanoric acid, orcin, erythrolein, azolitmin, spaniolitmint, flavonoids, saponins, tannins, glycosides, steroidal aglycone, carbohydrates, oligodynamic elements usnic acid, atronin, salazinic acid, tridecylmyristate, proto-lichesteric acid, 3-ketooleanane, icosan-1-ol, lecanoric acid, labdane type diterpenoid; named permelandone, and lano-2-en-type triterpene; named permelanostene [21,22,23,24].

Traditionally, *P. perlatum* has been used to relieve the pain of liver, stomach, renal, lumber region [21], and to manage the diarrhea, abdominal colic, dyspepsia, bronchitis, airway congestion, spermatorrhoea and dysentery [25,26,27,28,29]. *P. perlatum* possess the anti-diabetic, anti-hyperlipidemic, antiemetic, anti- hypertensive, antifungal, antiurolithiatic, cardiotonic, analgesic, wound healing, cytotoxic, and diuretic activities [21,30,31,32,33]. Furthermore, caperatic acid and diffractic acid, known constituents of lichens, exhibited the antispasmodic, analgesic, antiviral, antipyretic activities [26,34]. Moreover, usnic acid, an active constituent, exhibited antibacterial, antitumor [35,36], anti-mitotic [21,26], anticholinergic [37] and antioxidant [38,39] activities.

However, despite the traditional uses of *P. perlatum* for the treatments of airway, GIT, and cardiovascular problems, no study hasbeen reported with respect to its pharmacological effectiveness. Thus, current project was performed to authenticate the traditional uses of *P. perlatum,* and to investigate the possible underlying pharmacological mechanism via comparing with clinical drugs as a positive control.

## 2. Results

### 2.1. Phytochemical Analysis of Pp.Cr

**Figure 1:**
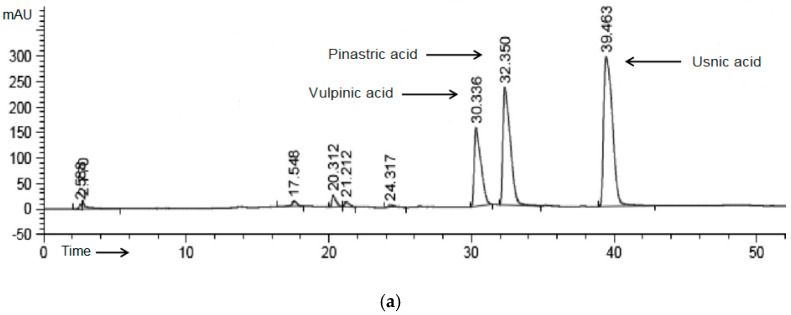

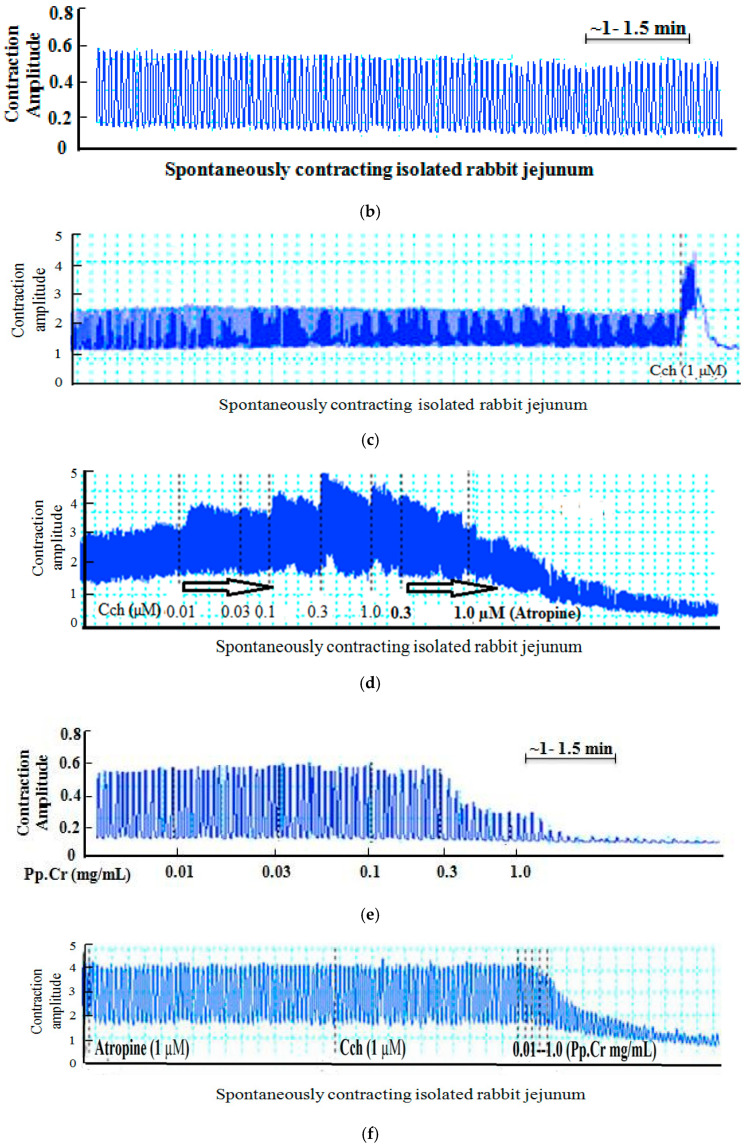

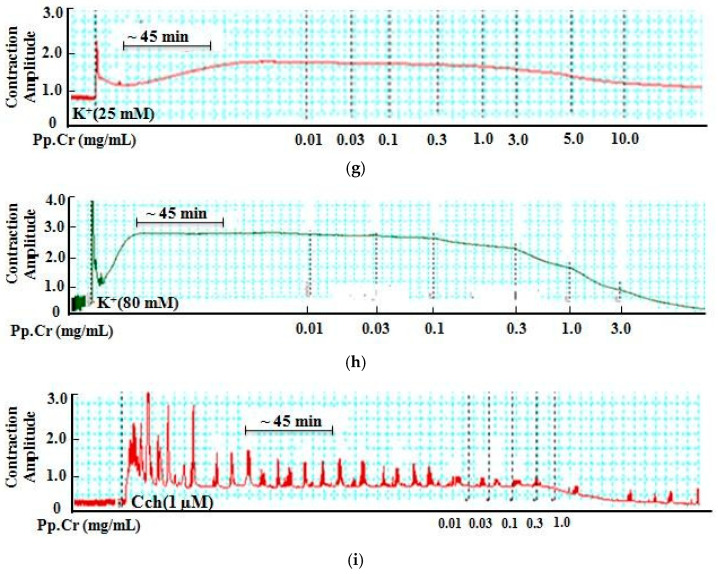
HPLC chromatogram acquired at 254 nm of the extract of *P. perlatum* (**a**). Tracing showing rhythmic spontaneously contracting isolated rabbit jejunum preparation (**b**), contracting effect of Cch (1 µM) on rabbit jejunum preparations (spasmogenic effect) (**c**), relaxant effect of atropine (0.3 and 1.0 µM) on Cch-induced (0.01, 0.03, 0.1, 0.3, and 1.0 µM) jejunum contraction (**d**), and relaxant effect of Pp.Cr (mg/mL) on spontaneously contracting jejunum with and without atropine (**e**,**f**), K^+^ (25 mM)- (**g**) K^+^ (80 mM)- (**h**) and Cch (1 µM)- (**i**) induced jejunum contractions.

Pp.Cr was found to have appreciable quantities of alkaloids and flavonoids while saponins, terpenes, tannins, sterols, and coumarins were moderately present. The results of phytochemical analysis of Pp.Cr are presented in Table S1. Moreover, the HPLC-UV technique provided excellent chromatographic peak resolution and baseline separation. (Figure 1a). Comparing the UV spectra (190–400 nm) and retention times (tR) with reference substances confirmed that Pp.Cr contains dibenzofuranusnic acid (tR = 39.463 min) and two pulvinic acid derivatives, pinastric acid (tR = 32.350 min), and vulpinic acid (tR = 30.336 min). The highest peak was shown by usnic acid (45.56 percent of the total area), followed by pinastric acid (30.54 percent) and vulpinic acid (vulpinic acid) (18.41 percent).

### 2.2. Acute Toxicity of Pp.Cr

Acute toxicity test uncovered that Pp.Cr did not show any mortality or changes in the behavioral or physical activities up to dose range of 6 gm/kg within 24 h in all test groups.The outcomes of acute toxicity of Pp.Cr are presented in Table S2.

### 2.3. Spasmolytic Effect of Pp.Cr

**Figure 2:**
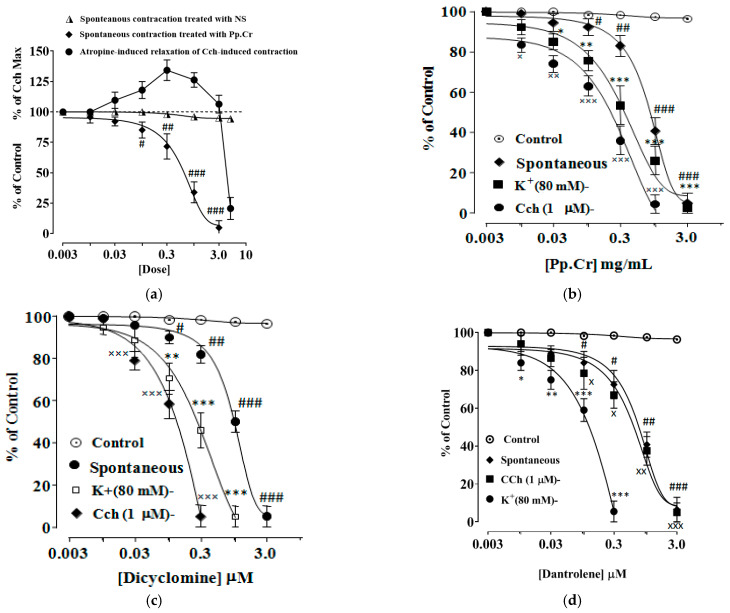
Concentration-dependent inhibitory effect of atropine (0.3 and 1.0 µM) and Pp.Cr (mg/mL) on Cch-induced contracting isolated rabbit jejunum preparations and spontaneously contracting rabbit jejunum preparations respectively (**a**). Concentration-dependent inhibitory effect of Pp.Cr (mg/mL) (**b**), dicyclomine (µM) (**c**) and dantrolene (µM) (**d**) against spontaneously contracting rabbit jejunum preparations, K^+^ (80 mM)- and Cch (1 µM)-induced jejunum contractions. ^#^ *p* < 0.05; ^##^ *p* < 0.01; ^###^ *p* < 0.001 compares the effects of various conc. of Pp.Cr (mg/mL) on spontaneous jejunum contractions. * *p* < 0.05; ** *p* < 0.01; *** *p* < 0.001 compares the effects of various conc. of Pp.Cr (mg/mL) on K^+^ (80 mM)-induced jejunum contractions. ^×^ *p* < 0.05; ^××^ *p* < 0.01; ^×××^ *p* < 0.001 compares the effects of various conc. of Pp.Cr (mg/mL) on Cch (1 µM)-induced jejunum contractions. (Mean ± SEM, *n* = 5).

Pp.Cr (0.01–1 mg/mL) demonstrated spasmolytic response on rhythmic spontaneously contracting isolated jejunum preparation with EC50 values of 1.15 mg/mL (0.85–1.62, 95% C.I, *n* = 5) (Figure 1a,b and Figure 2a). Spasmolytic response of Pp.Cr was same as dicyclomine and dantrolene (positive controls) with EC50 values 0.95 µM (0.7–1.3, 95% C.I, *n* = 5) and 1.1 µM (0.79–1.6, 95% C.I, *n* = 5), respectively (Figure 2b,c and Table S3). Pp.Cr did not show any response to low K^+^(25 mM)-induced contractions (Figure 1c). As expected, Pp.Cr relaxed the K^+^(80 mM)- and Cch(1 µM)-induced contractions with EC50 value of 1.96 mg/mL (1.66–2.28, 95% C.I, *n* = 5) and 0.55 mg/mL (0.13–2.15, 95% C.I, *n*= 5) (Figure 1d,e and Figure 2a) respectively. Dicyclomine revealed more potent response against Cch(1 µM)- than K^+^(80 mM)-induced contractions with respective EC50 value of 0.35 µM (0.05–1.96, 95% C.I, *n* = 5) and1.45 µM (0.55–2.98, 95% C.I, *n* = 5), as seen in Figure 2b. While dantrolene showed more effective response against K^+^(80 mM)- than Cch(1 µM)-induced with EC50 value of 0.28 µM (0.03–1.25, 95% C.I, *n* = 5) and 1.65 µM (0.65–3.15, 95% C.I, *n* = 5), respectively (Figure 2c).

**Figure 3:**
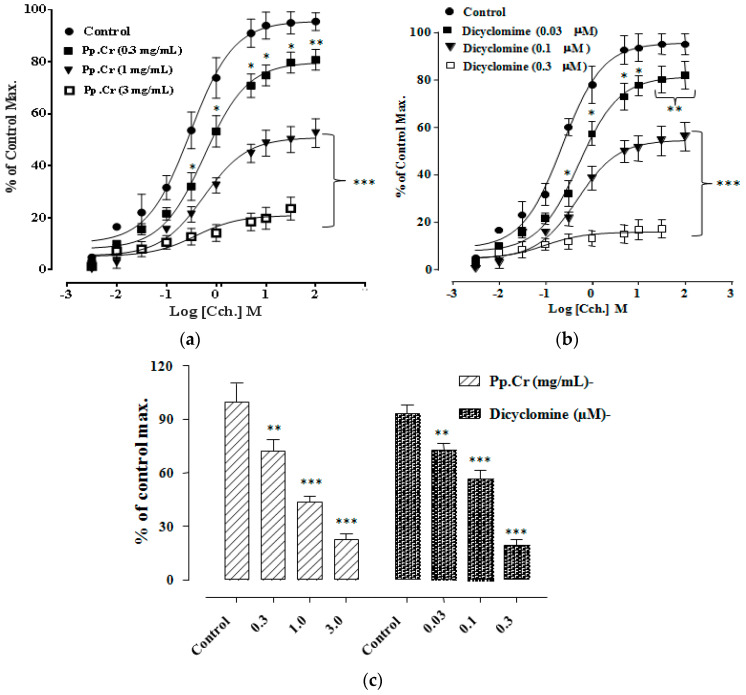
CRCs of Cch in the absence and presence of increasing conc. of Pp.Cr (mg/mL) (**a**) and dicyclomine (µM) on jejunum preparations (**b**) Statistical graph comparing CRCs of Cch in the absence and presence of Pp.Cr (mg/mL) and dicyclomine (µM) on jejunum preparations (**c**) * *p* < 0.05, ** *p* < 0.01, *** *p* < 0.001 compared to the respective control. (Mean ± SEM, *n* = 3).

**Figure 4:**
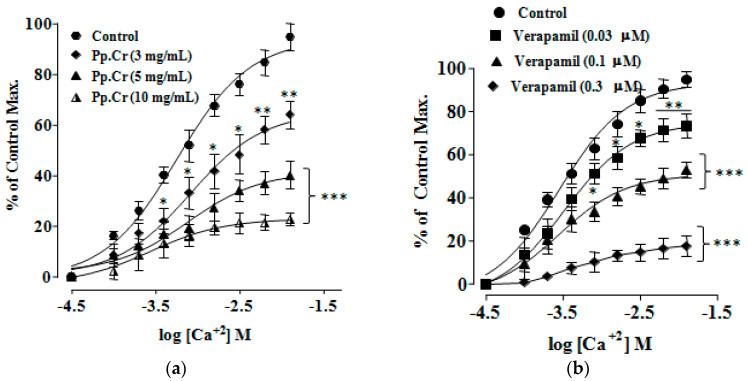

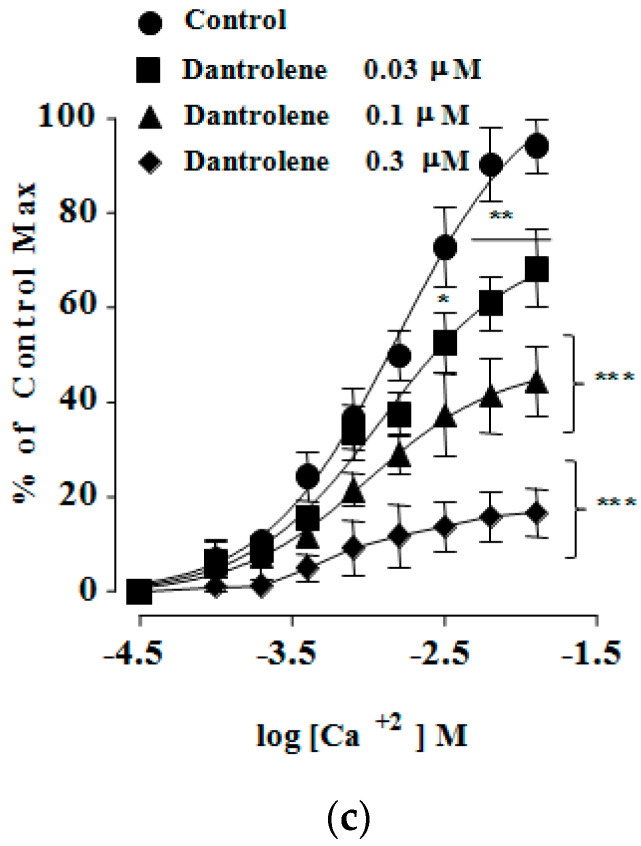
CRCs of Ca^+2^ in the absence and presence of increasing concentration of Pp.Cr (mg/mL) (**a**) verapamil (µM) (**b**) and dantrolene (µM) (**c**) on rabbit jejunum preparations. * *p* < 0.05, ** *p* < 0.01, *** *p* < 0.001 compared to the respective control. (Mean ± SEM, *n* = 3).

Pretreatment with Pp.Cr at 1.0 mg/mL and 3.0 mg/mL showed rightward non-parallel shift in CRCs of Cch (*p* < 0.001) (Figure 3a) whereas dicyclomine (0.1–0.3 µM) also showed similar pattern of CRCs of Cch shift (*p* < 0.001) (Figure 3b,c). When tested for involvement of Ca^+2^ channels, Pp.Cr (Figure 4a) and verapamil (0.03–0.3 µM) (Figure 4b) showed rightward shift in CRCs of Ca^+2^ (*p* < 0.001). Dantrolene (0.03–0.3 µM) also shifted the CRCs of Ca^+2^ in a similar pattern as expected (Figure 4c).

### 2.4. Tracheo-Relaxant Effect of Pp.Cr

**Figure 5:**
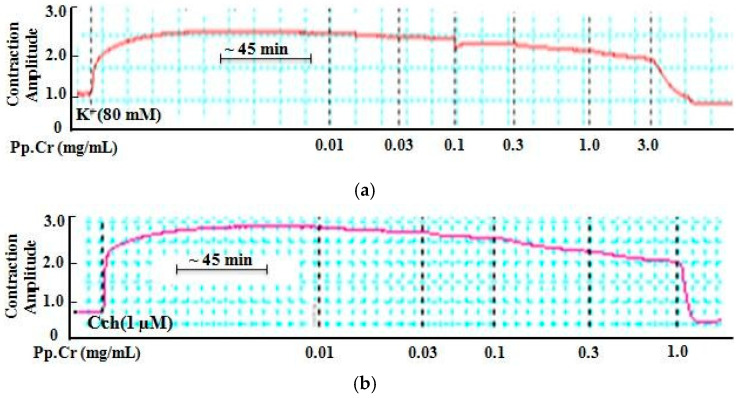

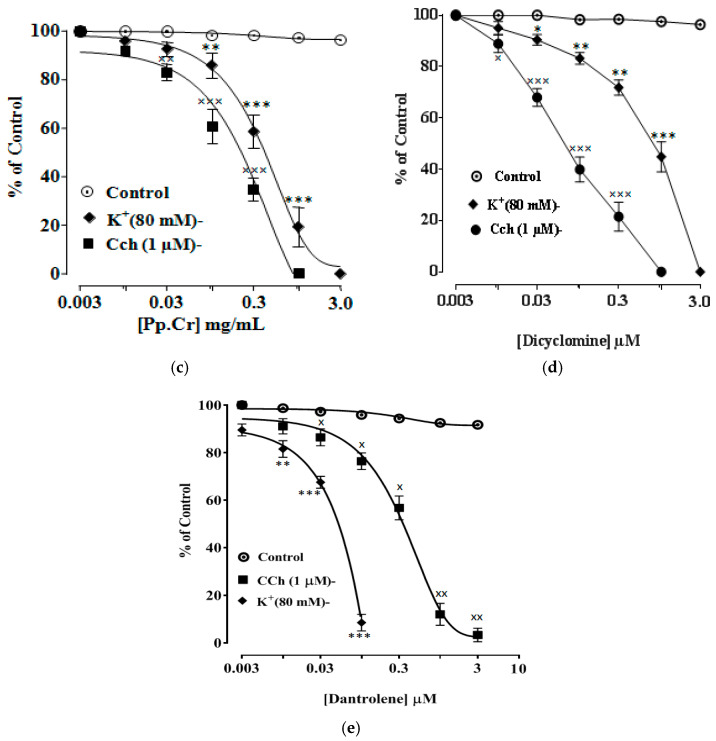
Concentration-dependent inhibitory effect of Pp.Cr (mg/mL) (**a**–**c**), dicyclomine (μM) (**d**) and dantrolene (μM) (**e**) against K^+^ (80 mM)- and Cch (1 µM)-induced trachea contractions. * *p* < 0.05, ** *p* < 0.01, and *** *p* < 0.001 compares the effects of various conc. of Pp.Cr (mg/mL)on K^+^ (80 mM)-induced trachea contractions. ^×^ *p* < 0.05, ^××^ *p* < 0.01, and ^×××^ *p* < 0.001 compares the effects of various conc. of Pp.Cr (mg/mL)on Cch (1 µM)-induced trachea contractions. (Mean ± SEM, *n* = 5).

**Figure 6:**
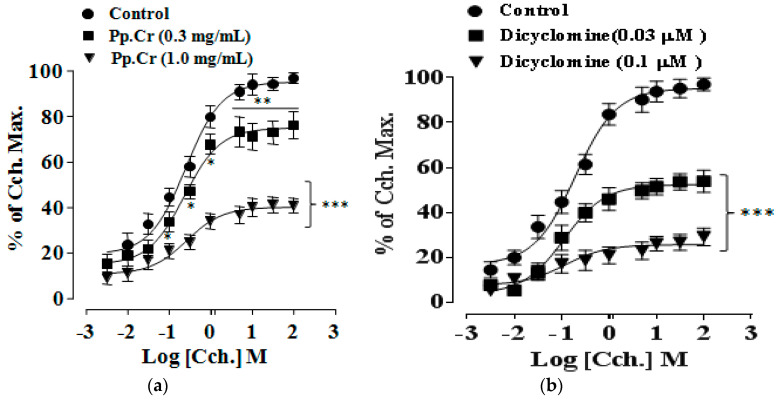
CRCs of Cch in the absence and presence of increasing concentration of Pp.Cr (mg/mL) (**a**) and dicyclomine (μM) (**b**) on rabbit trachea preparations. * *p* < 0.05, ** *p* < 0.01, and *** *p* < 0.001 compared to the respective control. (Mean ± SEM, *n* = 5).

Same as spasmolytic response, Pp.Cr demonstrated dose dependent relaxant effect on K^+^ (80 mM; 0.01–3 mg/mL)- and Cch (1 µM; 0.01–1 mg/mL)-induced tracheal contractions with EC50 value of 1.65 mg/mL (1.18–2.1, 95% C.I, *n* = 5) (Figure 5a,c) and 0.9 mg/mL (0.53–1.4, 95% C.I, *n* = 5) (Figure 5b,c), respectively, showed a more effective response against Cch. Dicyclomine and dantrolene also showed the similar inhibitory pattern against Cch (1 µM)- and K^+^ (80 mM)-induced contractions with an EC50 value of 0.6 µM (0.3–1.2, 95% C.I, *n* = 5) and 3.30 µM (2.39–4.54, 95% C.I, *n* = 5) (Figure 5d) and 4.15 µM (3.10–5.94, 95% C.I, *n* = 5) and 0.2 µM (0.03–1.3, 95% C.I, *n* = 5) respectively (Figure 5e).Same as CRCs of Cch of jejunum, Pp.Cr (0.3–1.0 mg/mL, *n* = 3) (Figure 6a), and dicyclomine (0.03–0.1 μM, *n* = 3) (Figure 6b) shifted the CRCs of Cch toward the right (*p* < 0.001).

### 2.5. Vasodilator Effect of Pp.Cr

**Figure 7:**
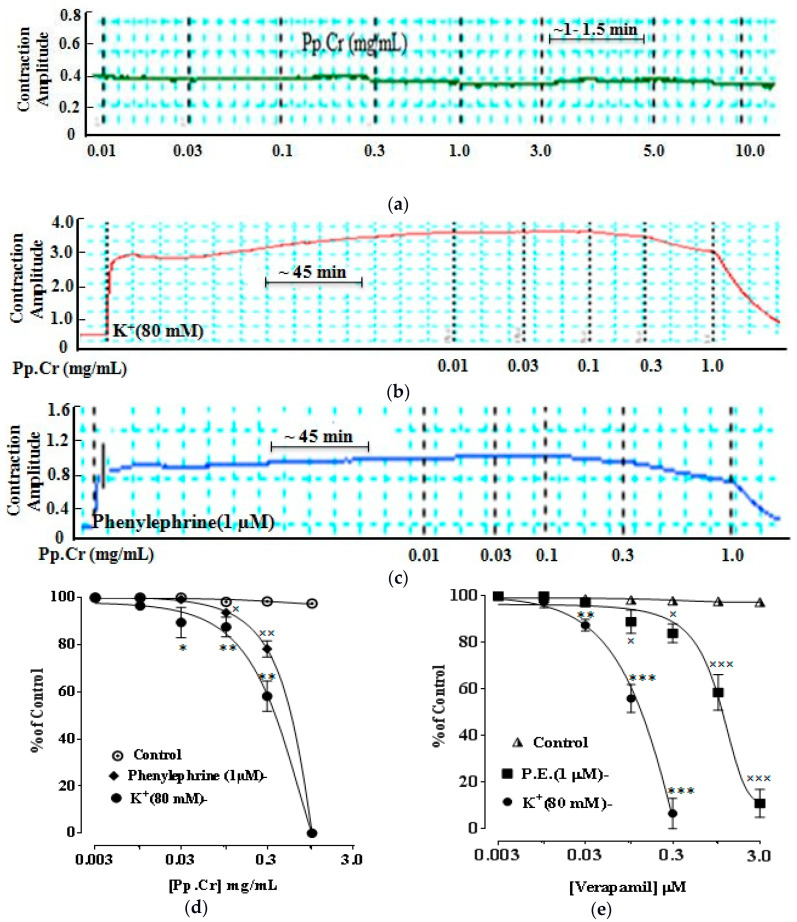

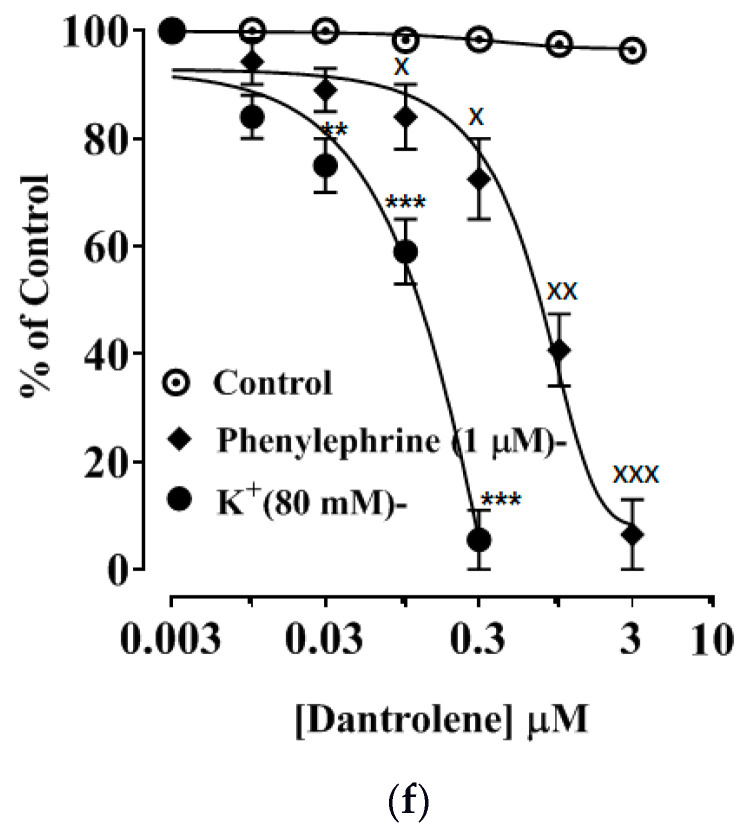
Tracing showing the effect of Pp.Cr (mg/mL) on un-stimulated rabbit aortic preparation as control (**a**). Concentration-response curves showing inhibitory effect of Pp.Cr (mg/mL) (**b**–**d**), verapamil (µM) (**e**) and dantrolene (µM) (**f**) against K^+^(80 mM)- and phenylephrine (1 µM)-induced aortic contractions. * *p* < 0.05, ** *p* < 0.01, and *** *p* < 0.001 compares the effects of various conc. of Pp.Cr (mg/mL) on K^+^ (80 mM)-induced aortic contractions. ^×^ *p* < 0.05, ^××^ *p* < 0.01, and ^×××^ *p* < 0.001 compares the effects of various conc. of Pp.Cr (mg/mL) on phenylephrine (1 µM)-induced aortic contractions. (Mean ± SEM, *n* = 5).

Pp.Cr (0.01–10.0 mg/mL) showed no response on stabilized rabbit aorta (Figure 7a). Nevertheless, Pp.Cr relaxed the K^+^(80 mM; 0.01–1 mg/mL)- and phenylephrine (1 µM; 0.01–1 mg/mL)-induced aortic contractions with EC50 values of 0.32 mg/mL (0.08–0.98, 95% C.I, *n* = 5) (Figure 7b,d) and 0.82 mg/mL (0.36–1.25, 95% C.I, *n* =5) (Figure 7c,d) respectively. Verapamil and dantrolene also revealed same inhibition pattern against K^+^(80 mM)- and phenylephrine (1 µM)-induced aortic contractions with EC50 values of 0.17 µM (0.022–0.46, 95% C.I, *n* = 5) and 1.98 µM (1.66–2.25, 95% C.I, *n* = 5) (Figure 7e) and 0.21 µM (0.039–0.66, 95% C.I, *n* = 5) and 2.3 µM (1.97–3.25, 95% C.I, *n* = 5) (Figure 7f) respectively.

## 3. Discussion

*P. perlatum* has been traditionally used for the treatment of diarrhea, bronchitis, asthma, airway congestion, and hypertension. Hence, this project was designed to authenticate the folkloric uses of *P. perlatum* as well as to investigate the possible basic mechanism(s) of action.

Methanolic extract from lichen *P. perlatum* (Pp.Cr) up to dose range of 6 gm/kg did not produce any behavioural changes, toxic effect and mortality within 24 h in all test groups. Keeping in view of folkloric uses of Pp.Cr, ethno-pharmacological study was conducted on rhythmic contracting jejunum preparations to evaluate underlying mechanism and possible effect. Pp.Cr showed antispasmodic response by decreasing both amplitude and frequency of the spontaneously contracting jejunum (Figure 1e). Jejunum’s contraction is mediated through activation of muscarinic M-3 receptors via stimulation of phospholipase C (PLC) and inositol triphosphate (IP3) followed by increased production of Ca^+2^ within cytosol either via Ca^+2^ discharge from sarcoplasmic supplies [40] or via Ca^+2^ influx from L-type channels. To evaluate the involvement of muscarinic receptors, carbachol, acetylcholine receptor agonist that mainly act onmuscarinicM3receptors, was tested on spontaneously contracting isolated rabbit jejunum preparations which exhibited the dose–dependent spasmogenic effect at the dose range of 0.01–1.0 µM while addition of atropine (anti-muscarinic agent) at the dose range of 0.3–1 µM revealed spasmolytic effect telling the involvement of muscarinic receptors (Figure 1c,d). The cholinergic mechanism (acetylcholine like) is thought to be involved in the spasmogenic action [11,15]. Next, spontaneous contracting preparations were pretreated with 1 µM of atropine, and allow stabilizing for 25 min. Afterward, stabilized jejunum preparations were further treated with Cch (1 µM) but tissues did not show the contacting effect. Next, tissues were rinsed with tyrode solution twice, allowed to acclimatize for 25 min, and further exposed to plant extract at the dose range of 0.01, 0.03, 0.1, 0.3, and 1 mg/mL which showed the spasmolytic effect, elucidating the involvement of muscarinic receptors (Figure 1f and Figure 2a). A similar pattern was followed for histamine, and histamine’s stimulatory action remained intact, suggesting that the impact is mediated through an acetylcholine-like mechanism (data not shown). This acetylcholine-like action was further confirmed when tissue was primed with pyrilamine (a histamine (H1) receptor blocker), which did not affect the acetylcholine response while totally blocking the histamine impact as predicted (data not shown). Acetylcholine regulates the peristaltic movement of the intestine via acting on the muscarinic (M3) receptor, whereas atropine (antagonist) inhibits the muscarinic receptors [41].

To investigate the possible mechanism, Pp.Cr was explored against Cch (1µM)-induced and K^+^(80 mM)-induced spastic contractions because contractions induced by Cch and high K^+^ are associated with muscarinic receptors activation and extracellular Ca^+2^ influx, respectively. As expected, Pp.Cr significantly relaxed Cch (1 µM)- and K^+^ (80 mM)-induced contractions, telling the blockade of muscarinic M-3 receptors and Ca^+2^ channels.Likewise, dicyclomine demonstrated same relaxant effect on rhythmic contracting jejunum preparations and on Cch (1 µM)-induced spastic contractions. Similar relaxant effect was showed by dantrolene against spontaneously contracting jejunum preparations as well as K^+^(80 mM)-induced contractions. Highlighting that spasmolytic activity of Pp.Cr is most probably due to blockade of both muscarinic receptors and Ca^+2^ channels.

In order to confirm the dual inhibitory effect of Pp.Cr, CRCs of Cch and Ca^+2^ were developed in the absence and presence of different concentration of Pp.Cr and standard drugs. Initially, Pp.Cr at decrease concentration (0.3 mg/mL) showed parallel shift of CRCs of Cch without suppression of maximum effect, revealing the involvement of a specific or competitive antagonist. Subsequently, Pp.Cr at next higher concentrations (1 and 3 mg/mL) revealed non-parallel rightward displacement (*p* < 0.001) demonstrating toward an additional non-competitive inhibition (Figure 3a). Similarly, dicyclomine (0.1 and 0.3 µM) caused a rightward non- parallel shift in the CRCs of Cch with a significant suppression (*p* < 0.001) (Figure 3b).

Pretreatment of jejunum with Pp.Cr shifted CRCs of Ca^+2^ rightward with a significant suppression (*p* < 0.001), confirming calcium channel blocking activity, but relatively at high concentration (3–10 mg/mL) as compared with CRCs of Cch (Figure 4a). Similarly, verapamil (0.1 and 0.3 µM; *p* < 0.001) (Figure 4b) and dantrolene (0.1 and 0.3 µM; *p* < 0.001) (Figure 4c) also demonstrated a rightward shift in the CRCs of calcium. Compiled data demonstrated that relaxant effect of Pp.Cr is mainly due to blockade of muscarinic receptors and calcium channels while antimuscarinic drugs and Ca^+2^ channels blockers are considered effective anti-motility, antidiarrheal, and antispasmodic agents. Thus, existence of dually acting spasmolytic activity of *P. perlatum* might be elaborating its significant effectiveness in abdominal spasm and diarrhea [12,17].

Based upon the folkloric use of *P. perlatum* to treat airway disorders, Pp.Cr was tested on Cch (1 µM)- and K^+^ (80 mM)-induced tracheal contraction. Pp.Cr at lower doses (0.01–1 mg/mL) significantly relaxed carbachol (1 µM)-induced contractions, but K^+^(80 mM)-induced contractions at a little higher concentrations (0.01–3 mg/mL), demonstrating the co-existence of antimuscarinic and calcium antagonist characteristics, same as dicyclomine. Bronchoconstriction occurs through activation of muscarinic receptors and Ca^+2^ channels because trachea is flooded with muscarinic receptors (M-1 and M-3) while muscarinic receptors antagonists are being used to treat bronchitis, asthma, and COPD [42]. Likewise, Ca^+2^ channel antagonists are used to treat airway disorders characterized by hyper-activation [43]. As expected, dicyclomine and dantrolene also demonstrated a similar pattern of relaxation against Cch (1 µM)- and K^+^ (80 mM)-induced tracheal contraction, respectively, signifying the dual mechanism of muscarinic antagonist and calcium channel blockade. These outcomes were further confirmed via constructing CRCs of Cch. Pp.Cr (0.3 mg/mL) (Figure 6a) and dicyclomine (0.03 and 0.1 µM) (Figure 6b) shifted the CRCs of Cch to the rightward (*p* < 0.001). Same as gut, parallel displacement of CRCs of Cch without suppression of maximum response at low dose suggested the presence of muscarinic receptors antagonism, whereas non-parallel shift with suppression of the maximum effect at high dose demonstrated the incidence of Ca^+2^ channel blockade. Pp.Cr was found somewhat more efficient against trachea than gut that may be either due to difference in involved physiological modulators [44,45], or because of synergistic interaction of the different inhibitory mechanism in airway compared to GIT [46], which cannot be ruled out. However, these significant effects authenticate the folkloric uses of *P. perlatum* to treat airways ailment like airway congestion, bronchitis, and asthma.

Additionally, *P. perlatum* relaxed both phenylephrine (1 µM)-induced and K^+^(80 mM)-induced aortic contraction, demonstrating Ca^+2^ antagonism because K^+^(80 mM)-induced aortic contraction are caused by activation and release of calcium either from endoplasmic reticulum or from L-type channels, while phenylephrine (1 µM)-induced contraction resulted in α-receptors activation and Ca^+2^ influx [47]. Verapamil and dantrolene also showed same relaxant response against both phenylephrine (1 µM)-induced and K^+^(80 mM)-induced aortic contraction showing that vasodilator effect is possibly due to Ca^+2^ channel blockade [17], thus providing the pharmacological basis for the traditional uses of *P. perlatum* to treat hypertension.

The phytochemical study of *P. perlatum* revealed the presence of flavonoids and alkaloids. Chromatograms obtained from HPLC-UV method exposed that usnic acid, an alkaloid, was the most abundant phytoconstituent in the extract.The observed antimuscarinic and Ca^+2^ antagonistic effects of the *P. perlatum* might be owing to presence of flavonoids and alkaloids, which have been previously reported to possess Ca^+2^ channel blocking [48], and anti-muscarinic [49] activities, respectively, but presence of other mechanisms and phytoconstituents cannot be neglected. This study also highlights the beliefs that natural products should be considered as a whole as they have multidimensional targets to deal with ailments along with possessing side effects neutralizing potentials [50].

## 4. Materials and Methods

### 4.1. Preparation of Crude Extract 

*P. perlatum* was purchased from the local pansar and was authenticated from expert taxonomist vide voucher #BZU.P.F1.709-11. Subsequent to collection, *P. perlatum* was rendered free from soil particles, adulterants and debris material through manual picking, and ground to crude powder (#40). For extraction process, triple maceration procedure was performed [51]. Approximately 1.5 kg of coarse powder was macerated with 70% aqueous-methanol in air tight amber glass bottle at room temperature, and agitated at rotary orbital shaker for 72 h at 120 rpm/min. Soaked, coarse powder was filtered and a dark brown thick extract named Pp.Cr, with estimated yield of 17.5%, was obtained as a result of rotary evaporation and lyophilization and stored at −4 °C for later use.

About 300 mg/mL (stock solution) was prepared by dissolving 0.3 gm of plant extract in 100 µL (0.1 mL) of 100% dimethyl sulfoxide (DMSO), which did not show any biological activity, to make volume up to 1000 µL (1 mL) with distilled water on the day of experiment, which was further subjected to serial dilutions to make 30 and 3 mg/mL.Extracted doses at the range of 0.01 mg/mL to 10 mg/mL (0.01, 0.03, 0.1, 0.3, 1, 3, 5, and 10 mg/mL) were used which are depicted in Table S4.

### 4.2. Chemicals and Standard Drugs 

Analytical grade standard drugs and chemicals were used. Carbachol (Cch), potassium chloride (KCl), magnesium chloride (MgCl_2_), verapamil hydrochloride, dicyclomine, ethylene diamine tetra-acetic acid (EDTA), dantrolene, and phenylephrine (PE) were purchased from Sigma Chemicals Co. St Louis, MO, USA. Difmethylsulfoxide (DMSO), glucose (C_6_H_12_O_6_), methanol (CH_3_OH), calcium chloride (CaCl_2_), magnesium sulphate (MgSO_4_), dichloromethane (DCM), potassium dihydrogen phosphate (KH_2_PO_4_), sodium bicarbonate (NaHCO_3_), and sodium dihydrogen phosphate (NaH_2_PO_4_) were obtained from Merck, Darmstadt, Germany. Sodium hydroxide (NaOH), ammonium hydroxide (NH_4_OH) and sodium chloride (NaCl) were acquired from BDH Laboratory supplies, Poole, England.

### 4.3. Animals

Animals (male/female) including thirty albino mice (4 to 6 weeks old and 35–50 gm) and twenty-five rabbits (1.0–1.8 kg, 6 to 7 months old) were bought from animal house of B.Z.U, Multan, and kept under controlled environmental condition (24 ± 3 °C; 40 to 60% humidity) with 12 h/12 h dark-light cycle. Acclimated animals had free access to distilled water and regular rodent chow. Ethical Committee B.Z.U, Multan (reference number. EC /04/2013, dated 4 September 2013) approved all procedures and experiments conducted in current study [52].

### 4.4. Phytochemical Screening

Phytochemical screening of plant extract was performed according to standard method to detect anthraquinones, alkaloids, tannins, terpenes, saponins, sterols, and flavonoids as possible important constituents [53].

### 4.5. HPLC System and Conditions

The dry plant extract was diluted in methanol to a concentration of 250 μg/mL before being tested. The HPLC system (Perkin Elmer^®^, Chicago, IL, USA) was connected to a Flexer Binary LC pump, UV/VIS LC Detector (Shelton CT^®^, Chicago, IL, USA), and reverse phase C18 column (5 mm, 250 × 4.6 mm) with an oven set at 30 °C for liquid chromatography.Chromera software was used to examine the data (version. 4.1.2.6410). The mobile phase was made up of 1% orthophosphoric acid in milli-Q water (A)/methanol (B), and the elution was done using a gradient technique [54]. A volume of 20 μL of sample was injected, with a flow rate of 0.6 mL/min and a temperature of 30 °C. The major peaks in the UV spectrum between 190 and 400 nm were scanned. Sigma Aldrich (St. Louis, MO, USA) provided the standards for this study.

### 4.6. Acute Toxicity Test

For possible assessment of behavioral (anorexia, mortality, gastric spasm, and diarrhea) and lethargic effect of plant extract, overnight fasted albino mice, with free access to distilled water, were grouped into five groups (6 in each). Mice of negative control group (group 1) were orally treated with 0.9% NaCl (normal saline) at dose range of 10 mL/kg while animals of test groups (group 2 to group 5) were orally treated with prepared extract at dose range of 1, 2, 4, and 6 gm/kg, respectively [16,49]. Animals were kept under keen supervision for 24 h after dosing for mortality and toxic effects like anorexia, gastrointestinal spasm, piloerection, and changes in exploratory behavior

### 4.7. Spasmolytic/Spasmogenic Activity on Isolated Rabbit Jejunum Preparations

Previously described methods were adopted for the assessment of spasmolytic/spasmogenic activity of plant extract [11]. After removing mesenteries and fecal masses, isolated jejunums were cut into segments of 2 cm and mounted in carbogen (5% CO_2_ and 95% O_2_) aerated tissue organ baths containing normal Tyrode’s solution aerated with carbogen (pH 7.4) at 37 °C. Prior to administration of plant extract and/or drugs to tissue organ baths in a cumulative fashion, hanged tissues were allowed to equilibrate. During experimentation, a tension of 1.0 gm was forced and isotonic contractions were recorded through isotonic force transducer (Model MLT0015, Colorado Springs, CO, USA). Under these experimental conditions, rhythmic spontaneously contracting isolated jejunum preparation were directly exposed to plant extract and standard drug, such as dicyclomine (dual blocker of muscarinic receptors and Ca^+2^ channels), for assessment of spasmolytic activity.

To investigate the Ca^+2^ channel blocking effect, stabilized jejunum tissues were first exposed to K^+^(80 mM) for 45 min to obtain sustained contraction because Ca^+2^ channels were speculated to be involved in spasmolytic activity [15], and then plant extract and dantrolene were separately applied in a cumulative manner for dose-dependent inhibitory responses through Ca^+2^ channels blockade [17]. To explore the involvement of any other spasmolytic effect, carbachol (Cch; is a parasympathomimetic that mimics the effect ofacetylcholineon both the muscarinic and nicotinic receptors)was applied at the dose of 1 µM to stabilize the tissues for 45 min to obtain Cch-induced contractions through activation of muscarinic receptors [55]. After achieving Cch-induced contractions, plant extract and dicyclomine were separately added in a cumulative manner for dose-dependent relaxant effect.

For authentication of Ca^+2^ channels antagonistic response, stabilized jejunum preparations were exposed to K^+^ (80 mM) in normal Tyrode’s solution for 40 min. Afterward, stabilized tissue was further exposed to K^+^-rich and Ca^+2^-free Tyrode’s solution for removal of Ca^+2^ from the jejunum tissues. The CRCs of Ca^+2^ were developed in the presence of different concentrations of the extract and standard drugs (verapamil and dantrolene) to assess a possible Ca^+2^ channel blocking effect. Shifting of CRCs of Ca^+2^ towards right in the presence of extract and standard drugs (verapamil, and dantrolene) in concentration-dependent manner confirms the blocking of Ca^+2^ channel activity. Finally, CRCs of Cch were constructed [56,57].

### 4.8. Bronchodilator Activity on Isolated Rabbit Tracheal Preparations

For the evaluation of bronchodilator effect, previously described protocols were followed [43]. For this purpose, fatty debris free trachea tubes comprising 2 to 3 mm rings and two cartilages were hanged in carbogen aerated tissue organ baths containing Krebs’s solution (C_6_H_12_O_6_ (11.7 mM), NaCl (118.2 mM), CaCl2 (2.5 mM), KH_2_PO_4_ (1.3 mM), NaHCO_3_ (25.0 mM), MgSO4 (1.2 mM) and KCl (4.7 mM)) of pH 7.4 at 37 °C. A preload tension of 1.0 gm was constantly applied during the experimental procedure. Before the application of test materials, mounted trachea tubes were stabilized for 45 minwith replacing Kreb’s solution after every 15 min. The isometric contractions were recorded through force transducers coupled with Power lab data acquisition system (AD Instruments, Sydney, Australia) attached to a computer installed with lab chart software (version 6.0). Afterward, stabilized trachea tubes were exposed to Cch (1 µM) and K^+^(80 mM) for 45 min to obtain persistent contraction, and then plant extract, dicyclomine and dantrolene were separately applied in a cumulative manner for assessment of possible relaxant effect and possible mechanism of action. For confirmation of underlying mechanism of action, CRCs of Cch were constructed in the presence and absence of various concentrations of dicyclomine and extract [55].

### 4.9. Vasodilator Activity on Isolated Rabbit Aorta Preparations

To evaluate the vasodilator effect, isolated rabbit aorta preparations (approximately 2–3 mm broad) were hanged in carbogen aerated tissue organ baths containing Krebs’s solution with a consistent preload tension of 2 gm. Mounted aortic preparations were stabilized for 45 min prior to application of plant extract and/or drugs, during which Kreb’s solution was replaced after every 15 min [13]. Afterwards, vasodilator effect was noted by cumulative application of plant extract to pre-exposed K^+^ (80 mM) and phenylephrine (1 µM) induced aortic preparations. Finally, verapamil and dantrolene were separately added to K^+^(80 mM)-induced and phenylephrine (1 µM)-induced aortic preparations to confirm the possible underlying mechanism.

### 4.10. Statistical Analysis

Data was presented as mean ± SEM (standard error mean). EC_50_ values were expressed with 95 percent confidence intervals (95% C.I). Graph pad Prism version 6.0 was used to plot the logarithmic dose-response curves. One-way ANOVA (analysis of variance) was used and *p* < 0.05 was considered statistically significant.

## 5. Conclusions

It can be inferred that spasmolytic and bronchodilator effects of *P. perlatum* may be related to dual blockade of muscarinic receptors and Ca^+2^ channels whereas vasodilator effect may be due to Ca^+2^ channels blockade. The presence of usnic acid, an alkaloid, might explain the antimuscarinic and Ca^+2^ antagonistic actions of *P. perlatum.*. Hence, present study is providing solid mechanistic background to validate the folkloric uses of *P. perlatum* to treat diarrhea, airway congestion, bronchitis, and hypertension.

## Data Availability

Available data are presented in the manuscript.

## Supplementary Materials

Table S1: Data obtained after the phytochemical analysis; Table S2: Acute toxicity study of Pp.Cr; Table S3: Obtained EC50 values of plant extract; Table S4: (a) Obtained EC50 values of plant extract; (b) Dosing protocols of positive drugs (standard drugs).

## Sample Availability

Samples of the compounds are available from the authors.
