# Supplementary material for: The Spasmolytic, Bronchodilator, and Vasodilator Activities of Parmotrema perlatum Are Explained by Anti-Muscarinic and Calcium Antagonistic Mechanisms
Source: Molecules. 2021 Oct 20;26(21):6348. doi: 10.3390/molecules26216348 (PMC8588472; doi:10.3390/molecules26216348)
Supplement: Supplementary file 1 [file molecules-26-06348-s001.zip › molecules-1397479-Supplementary Materials-new version.pdf]

Sr.no	Test Performed	Results	Symbolic representation
1	Alkaloid	Positive	+ +
2	Flavonoids	Positive	+ +
3	Saponins	Positive	+
4	Tanins	Positive	+
5	Terpenes	Positive	+
6	Sterols	Positive	+
7	Anthraquinones	Negative	-

Table S1. Data obtained after the phytochemical analysis.

S. No	Response	Animals				
		Before treatment	After treatment (1 g/kg)	After treatment (2 g/kg)	After treatment (4 g/kg)	After treatment (6 g/kg)
1	**Alertness**	Normal	Normal	Normal	Normal	Normal
2	**Grooming**	Absent	Absent	Absent	Absent	Absent
3	**Restlessness**	Absent	Absent	Absent	Absent	Absent
4	**Touch response**	Normal	Normal	Normal	Normal	Normal
5	**Gastrointestinal spasm**	Absent	Absent	Absent	Absent	Absent
6	**Food intake**	Normal	Normal	Normal	Normal	Normal
7	**Water intake**	Normal	Normal	Normal	Normal	Normal
8	**Mortality**	N/A	Nil	Nil	Nil	Nil
9	**Urination**	Normal	Normal	Normal	Normal	Normal
10	**Salivation**	Normal	Normal	Normal	Normal	Normal
11	**Piloerection**	Absent	Absent	Absent	Absent	Absent

Table S2. Acute toxicity study of Pp.Cr.

Part used	Treatments	EC_{50}
Rabbit jejunum preparation	Pp.Cr (0.01–1 mg/mL) relaxed rhythmic spontaneous contraction	1.15 mg/mL
	Dicyclomine relaxed rhythmic spontaneous contraction	0.95 µM
	Dantrolene relaxed rhythmic spontaneous contraction	1.1 µM
	Pp.Cr (0.01–1 mg/mL) relaxed K^+(80 mM)- induced contractions	1.96 mg/mL
	Pp.Cr (0.01–1 mg/mL) relaxed Cch(1µM)-induced contractions	0.55 mg/mL
	Dicyclomine relaxed Cch(1µM)-induced contractions	0.35 µM
	Dicyclomine relaxed K^+(80 mM)-induced contractions	1.45 µM
	Dantrolene relaxed Cch(1µM)-induced contractions	1.65 µM
	Dantrolene relaxed K^+(80 mM)-induced contractions	0.28 µM
Rabbit tracheal preparation	Pp.Cr (0.01–3 mg/mL) relaxed K^+(80 mM)- induced contractions	1.65 mg/mL
	Pp.Cr (0.01–1 mg/mL) relaxed Cch(1µM)-induced contractions	0.9 mg/mL
	Dicyclomine relaxed Cch(1µM)-induced contractions	0.6 µM
	Dicyclomine relaxed K^+(80 mM)-induced contractions	3.30 µM
	Dantrolene relaxed Cch(1µM)-induced contractions	4.15 µM
	Dantrolene relaxed K^+(80 mM)-induced contractions	0.2 µM
Rabbit aorta preparation	Pp.Cr (0.01–1 mg/mL) relaxed K^+(80 mM)- induced contractions	0.32 mg/mL
	Pp.Cr (0.01–1 mg/mL) relaxed phenylephrine (1 µM) induced contractions	0.82 mg/mL
	Verapamil relaxed K^+(80 mM)- induced contractions	0.17 µM
	Verapamil relaxed phenylephrine (1 µM) induced contractions	1.98 µM
	Dantrolene relaxed K^+(80 mM)- induced contractions	0.21 µM
	Dantrolene relaxed phenylephrine (1 µM) induced contractions	2.3 µM

Table S3. Obtained EC50 values of Plant extract

Table S4. Dosing protocols of plant extract.

Extract	Cumulative	Bolus	Cumulative	Bolus	Cumulative	Bolus	Cumulative	Bolus	Mg/ml
3	17 µl	17 µl	33 µl	33 µl	50 µl	50 µl	66 µl	66 µl	0.01
	33 µl	50 µl	66 µl	100 µl	99 µl	150 µl	132 µl	200 µl	0.03
30	12 µl	17 µl	23 µl	33 µl	36 µl	50 µl	46 µl	66 µl	0.1
	33 µl	50 µl	66 µl	100 µl	99 µl	150 µl	132 µl	200 µl	0.3
300	12 µl	17 µl	23 µl	33 µl	36 µl	50 µl	46 µl	66 µl	1
	33 µl	50 µl	66 µl	100 µl	99 µl	150 µl	132 µl	200 µl	3
	33 µl	83 µl	66 µl	166 µl	99 µl	250 µl	132 µl	266 µl	5
	83 µl	166 µl	166 µl	333 µl	250 µl	500 µl	333 µl	666 µl	10

Dosing protocols of positive drugs (standard drugs)

Stock solution	5 ml (Bath)	10ml (Bath)	15 ml (Bath)	20 ml (Bath)	Final bath Conc.
Ach (10^{-4})	15 µl	30 µl	45 µl	60 µl	$3x10^{-7}$ (0.3µM)
Cch (10^{-4})	50 µl	100 µl	150 µl	200 µl	1 µM
PE (10^{-4})	50 µl	100 µl	150 µl	200 µl	1 µM
KCl (2.5M)	160 µl	320 µl	480 µl	640 µl	80mM
